# Bridging Differences in Outcomes of Pharmacoepidemiological Studies: Design and First Results of the PROTECT Project

**DOI:** 10.2174/1574884708666131111211802

**Published:** 2014-05

**Authors:** Victoria Abbing-Karahagopian, Xavier Kurz, Frank de Vries, Tjeerd P. van Staa, Yolanda Alvarez, Ulrik Hesse, Joerg Hasford, Dijk Liset van, Abajo Francisco J. de, John G. Weil, -Bensouda Lamiae Grimaldi, Antoine C.G. Egberts, Reynolds Robert F., Klungel Olaf H.

**Affiliations:** 1 Utrecht Institute for Pharmaceutical Sciences (UIPS), Division of Pharmacoepidemiology and Clinical Pharmacology, Utrecht University, Utrecht, The Netherlands;; 2 European Medicines Agency (EMA), London, United Kingdom;; 3 CAPHRI, School for Public Health and Primary Care, Maastricht University, The Netherlands;; 4 Clinical Practice Research Datalink, Medicines and Healthcare Products Regulatory Agency, London, United Kingdom;; 5 National Institute for Health Data and Disease Control, Sector for National Health Documentation and Research, Copenhagen, Denmark;; 6 Institute for Medical Informatics, Biometry and Epidemiology, Ludwig-Maximilians-Universität, Munich, Germany;; 7 NIVEL, Netherlands Institute for Health Services Research, Utrecht, The Netherlands;; 8 BIFAP Research Unit (Spanish Agency for Medicines and Medical Devices) and University of Alcalá, Madrid, Spain;; 9 GlaxoSmithKline R&D, Harlow, UK;; 10 LA-SER & Pasteur Institute (Pharmacoepidemiology and Infectious Diseases Unit), Paris, France;; 11 University Medical Center Utrecht (UMCU), Department of Clinical Pharmacy, Utrecht, The Netherlands;; 12 Epidemiology, Pfizer, New York, NY, USA;; 13 University Medical Center Utrecht (UMCU), Julius Center for Health Sciences and Primary Care, The Netherlands

**Keywords:** Adrenergic beta-agonists, anti-bacterial agents, anticonvulsants, antidepressive agents, benzodiazepines, bone, calcium channel blockers, drug toxicity, european medicines agency, fractures, liver injury, myocardial infarction, neoplasms, observational studies, pharmacoepidemiology, suicide.

## Abstract

**Background:**

Observational pharmacoepidemiological (PE) studies on drug safety have produced discrepant results that may be due to differences in design, conduct and analysis.

**Purpose:**

The pharmacoepidemiology work-package (WP2) of the Pharmacoepidemiological Research on Outcomes of Therapeutics by a European ConsorTium (PROTECT) project aims at developing, testing and disseminating methodological standards for design, conduct and analysis of pharmacoepidemiological studies applicable to different safety issues using different databases across European countries. This article describes the selection of the safety issues and the description of the databases to be systematically studied.

**Methods:**

Based on two consensus meetings and a literature search, we selected five drug-adverse event (AE) pairs to be evaluated in different databases. This selection was done according to pre-defined criteria such as regulatory and public health impact, and the potential to investigate a broad range of methodological issues.

**Results:**

The selected drug-AE pairs are: 1) inhaled long-acting beta-2 agonists and acute myocardial infarction; 2) antimicrobials and acute liver injury; 3) antidepressants and/or benzodiazepines and hip fracture; 4) anticonvulsants and suicide/suicide attempts; and 5) calcium channel blockers and malignancies. Six European databases, that will be used to evaluate the drug-AE pairs retrospectively, are also described.

**Conclusion:**

The selected drug-AE pairs will be evaluated in PE studies using common protocols. Based on consistencies and discrepancies of these studies, a framework for guiding methodological choices will be developed. This will increase the usefulness and reliability of PE studies for benefit-risk assessment and decision-making.

## INTRODUCTION

Randomised Clinical Trials (RCT) of drug adverse events do not optimally reflect real life situations: small sample sizes, highly selected populations and short duration of exposures [1]. During the past decades, it has been realized that adverse drug-events (AE) need to be further evaluated in pharmacoepidemiological (PE) studies [2]. PE methods were, however, still in development and therefore had the potential for reporting biased results. An example is the falsely reported relationship of breast cancer to use of the blood pressure lowering drug reserpine [3]. The growing availability of large routine electronic health record databases has made it possible to study less frequent and less severe AEs. An example is the risk of deep venous thrombosis in users of third generation oral contraceptives [4]. Alhough (pharmaco)-epidemiological methods have progressed, the challenge of studies of low absolute and relative risks associated with medications may have pushed pharmacoepidemiology to the borders of what can reliably be detected beyond the level of background noise [5]. Furthermore, efforts focusing on evaluation of type A AEs (those with dose dependent and predictably augmented pharmacological effects) and intended effects of drugs have increased the potential for bias [6]. 

Study conduct and design choices are one of the factors contributing to the diversity and discrepancy of study results. For instance, using the same database (the UK Clinical Practice Research Datalink [7] and including a large number of patients, two studies that were independently conducted reached very different conclusions [8, 9]. Within the same source study population, discrepant results between studies can be explained by small differences in study design such as different definitions of exposure time windows, confounder selection and age matching [9, 10]. Moreover, exposure-time-dependent hazard functions can substantially affect comparisons between different studies of the same drug [11]. The use of different statistical methods to adjust for confounding is another explanation for dissimilar study results [12]. For instance, in a database study and in simulation studies, systematic differences were found in effect estimates when propensity scores were used compared to logistic regression or Cox-proportional hazards regression [13, 14]. Immortal time bias has been suggested as another important source of variability in results between observational studies on drug effects [15]. Furthermore, several studies that have evaluated the same data source have drawn different conclusions about the plausibility of a pharmacological explanation of an observed association. Among these are: use of inhaled corticosteroids and risk of hip fracture [16, 17], use of beta-blocker and risk of hip fracture [18, 19]; use of oral bisphosphonates and risk of cancer of oesophagus [20, 21]; and more recently, use of proton pump inhibitors and risk of hip fracture [22-25].

The influence of methodological variation should be minimized and quantified, in order to interpret differences in associations between drugs and AEs that arise between types of data sources and healthcare systems in the different countries. A clear interpretation of differences in results between studies performed in the same database, and between different databases, is currently not completely feasible due to these methodological differences. This situation poses difficulties for all stakeholders, such as regulatory agencies, industry, healthcare professionals and patients. Difficulties in interpreting individual and/or groups of observational studies limit their usefulness for decision making on the benefit-risk balance of drugs. These experiences highlight the need to increase understanding of the implications of different methodological choices by investigators and for a framework on PE methodology across different data sources. To understand and subsequently validate differences caused by methodological and non-methodological (data related) factors we have selected five different drug-AE pairs, to be analysed in five different European databases based on a common protocol that includes extensive sensitivity analyses.

The Pharmacoepidemiological Research on Outcomes of Therapeutics by a European ConsorTium (PROTECT) study is a collaborative European project that addresses limitations of current methods in the field of pharmacoepidemiology and pharmacovigilance [26]. PROTECT is a multinational consortium of 29 partners including academics, regulators, small and medium enterprises (SMEs) and European Federation of Pharmaceuticals Industries and Associations (EFPIA) companies, coordinated by the European Medicines Agency (EMA) with GlaxoSmithKline (GSK) as deputy co-ordinator. The “Framework for pharmacoepidemiology studies” work-package (WP2) of PROTECT, co-led by Utrecht University and Pfizer, aims at developing, testing and disseminating methodological standards for the design, conduct, and analysis of PE studies applicable to different safety issues using different data sources. This article presents the rationale, design and the first results of the WP2 of PROTECT initiative. 

## METHODS 

## Selection of Drug-AE Pairs

Criteria for the selection of key AEs to evaluate in different databases included: 1) the AE selected having resulted in (major) regulatory decisions such as drug withdrawal or major summary of product characteristics (SmPC) changes; 2) public health impact aspects including seriousness of the event (prioritise more serious events); having variable incidence rates (both rare and common events); and prevalence of drug exposure (commonly used drugs and infrequently used drugs); 3) possibility to investigate a broad range of relevant methodological issues including feasibility to ascertain events in electronic healthcare databases (events both easy and difficult to ascertain); hazard functions (acute and long-term effects, delayed/transient effects); setting of drug use (in-/outpatient use); type of use (short/long-term, as needed); and different indications of use. All drug-AE pairs needed to fulfil these criteria. Furthermore, at least one drug-AE pair was selected taking into account those chosen by the public-private US initiative Observational Medical Outcomes Partnership (OMOP) in order to facilitate comparison with this initiative [27].

An initial inventory of potential drug-AE pairs was compiled, based on recommendations from public and private partner experts in the field of epidemiology and pharmacovigilance (European and national medicines agencies, pharmaceutical industry and academia). All partners were asked to nominate 10 drug-AE pairs that would fulfil the previously defined criteria for selection. This resulted in an initial list of 55 AEs and >55 individual drugs and drug classes. A first consensus meeting produced five AEs and a limited number (≤3) of drugs per AE with high priority. Supported by extensive research of the scientific literature and publicly available information sources, including PubMed, EMA and the US Food and Drug Administration (FDA) websites, each of the criteria for the selected drug-AE pairs was assessed. Subsequent to this assessment, the selection of five drug-AE pairs was finalized in a second consensus meeting. 

## Databases

All PROTECT partners who manage or have access to electronic healthcare or reimbursement databases were asked to describe characteristics of these databases. Databases incorporated medical and registry-based data sources, such as the Danish national registries, the Dutch Mondriaan project, the British CPRD and The Health Improvement Network (THIN) databases, the Spanish BIFAP project and the German Bavarian claims database. In addition, the French PGRx case-referent system will be made available to investigate and/or confirm some of the drug-AE pairs. All partners were sent a questionnaire in order to systematically collect the information. Parameters included information on period of data collection, coding systems, accessibility procedures and an extensive list of specific categories for longitudinally collected data such as drug prescribing/dispensing, clinical data, laboratory test data and life style parameters. The databases from the Netherlands, Spain, Denmark, and UK are based on primary care (GP and/or Pharmacy) covering all prescription drugs regardless of reimbursement.

## Analytical Approach

Common study protocols to study each of the drug-AE pairs have been developed and comply with the ENCePP methodological standards (including the ENCePP checklist) and were submitted to the ENCePP registry of studies [28]. These protocols include different study designs such as cohort, case-control, and case-cross-over design. All studies are retrospective, based on existing data from the databases described above. We will use data from the period 2001-2009. Inclusion for entry in the cohort studies is that subjects would have to have at least 1 recorded prescription or dispensing of the drug of interest. This approach reduced confounding by indication and still allows comparing between subjects that are on the drug at a certain time during follow-up versus subjects that are not currently on the drug but used the drug in the past. Operational definitions of exposures and outcomes are harmonized as much as possible and varied in a range that reflects the possibilities and limitations of the available databases. For the outcome of liver injury a automated algorithm has been developed taking into account diagnostic codes and laboratory tests. Detailed code lists are available upon request. Exposure will be analysed time-dependently in all studies and some confounders will also be classified time-dependently if appropriate. Different methods for the selection of and control for confounding variables will be applied. Not all databases have the same level of detail with regard to confounders. We will conduct an analysis for each drug-ae pair that includes a minimum set of confounders that all databases have available. In subsequent sensitivity analyses we will also assess the impact of further adjustment for confounders that are available in some, but not all databases. For all databases we will describe exposure to the drugs of interest and for those databases with sufficient information on diagnoses we will describe the outcomes of interest. For the association studies we have implemented a blinding procedure with central results management. Results for each design will be un-blinded only after all databases have been analysed and produce the adjusted association measures.

## RESULTS

## The Drug-AE Pairs 

The five drug-AE pairs fulfilling the a priori defined criteria are: 1) inhaled long-acting beta-2 agonists and acute myocardial infarction; 2) antimicrobials and acute liver injury; 3) antidepressants and/or benzodiazepines and hip fracture; 4) anticonvulsants (approved for treatment of epilepsy) and suicide/suicide attempts; 5) calcium channel blockers and malignancies. The following information is described for each drug-AE pair: public health impact, drug utilisation, the level of evidence to support a causal association, the proposed pharmacological mechanism(s), and methodological challenges specific for the drug-AE association. Table **1** shows the selected AEs and their characteristics. Table **2** shows the characteristics of the selected drugs. Table **3** displays the drug-AE associations and characteristics such as the range of relative risks, the study designs that have been used to study the association, the main methodological issues, and the suggested hazard function (in relation to onset and offset of the increased risk after initiation or discontinuation of the drug).

## The Databases

General features of the databases participating in PROTECT are presented in Table **4**. The six databases contain data from patients from five different European nations: the Danish national registries, the Dutch Mondriaan database, the British CPRD and THIN databases, the Spanish BIFAP project, the German Bavarian claims database. The Danish registries have national coverage, while other databases contain regional data or a representative sample of a total population. All the databases were quite representative of their nation. Most of the databases were established more than 10 years ago with regular and expanding data collection and validation history. Routine checks on quality are performed in all databases. The majority of databases include GP data and two (Danish and CPRD) include registries for and linkages to mortality, cancer, and secondary care data. Three (Danish registries, Mondriaan and Bavarian claims) out of six databases include or had linkages to claims data. A particular characteristic of the Bavarian Claims database is the availability of information on prescriptions and diagnoses in quarters of a calendar year. The exact dates of prescribing and diagnoses are not available. Therefore, we decided to use this database for descriptive purposes only and refrained from conducting association studies for which this information is pivotal. For some databases, linkage to other national registries requires additional procedures and financial compensation. Table **4** briefly describes the participating databases. 

## DISCUSSION AND UPCOMING STUDIES IN PROTECT

We prioritised five drug-AE associations that are highly relevant from the perspective of various stakeholders including regulatory agencies, patients and the pharmaceutical industry. These associations allow investigation of the influence of variation in methodology. Furthermore, we characterised seven routine electronic healthcare databases from five European countries that will be used for the evaluation of the selected drug-AE associations.

The work of WP2 of PROTECT is in the front line of currently on-going large (inter-) national initiatives such as the Observational Medical Outcomes Partnership (OMOP), FDA Sentinel Initiative [29] and EU-ADR (EU-Adverse Drug Reactions) project [30]. OMOP is a public-private partnership that conducts experiments to assess value, feasibility, and utility of observational data to identify and evaluate the safety risks and potential benefits of prescription drugs [31]. Furthermore, OMOP tests approaches for creating the infrastructure for accessing and managing the required data. The FDA Sentinel initiative aims at development of a national electronic safety monitoring system in order to strengthen FDA’s ability to monitor post-marketing performance of medical products and to enable FDA to access existing automated healthcare data by partnering with data holders. EU-ADR project is focussing on utilizing electronic healthcare data records and biomedical databases for the early detection of AEs. In the EU-ADR project a list of 23 events were judged as important in pharmacovigilance and three AE (acute myocardial infarction, acute liver injury, and suicidal behaviour/attempt) on this list have also been prioritised in our project. The OMOP project has also defined a list of health outcomes of interest (HOI) and drug pairs to be further investigated. As previously mentioned we included two of these pairs (DILI and antimicrobials, hip fracture and benzodiazepines) in our prioritised list of five drug-AE pairs. Although these projects have a different focus than those of WP2 of PROTECT, the overlap in prioritised AEs (and drugs) will facilitate comparisons. 

The strengths of our approach include the development of a common study protocol (that includes variation in methodology e.g. different designs) for five drug-AE associations that will be studied in different databases. In addition, some of our findings will be confirmed in specific registries such as PGRx [32]. Our approach will allow us to distinguish between variation in results due to variation in methodology and those due to database differences. Analysing these discrepancies will provide guidance regarding the optimal methodology for certain safety issues and the optimal selection of appropriate data source(s). The experience obtained in the PROTECT database network will improve the possibilities for multinational database studies for various safety issues, including the investigation of rare serious AE. Finally, other research activities of WP2 of PROTECT will further improve the methodological guidance on pharmacoepidemiological studies. These include an evaluation and improvement of methods to control for confounding such as propensity scores and instrumental variables in simulation studies, and drug utilisation research. 

A limitation of our approach may be the scope of the drug-AE pairs and selected healthcare databases. Our findings may not be extendable to other safety issues or other databases that we do not study. However, our selection of drug-AE pairs includes common drug safety issues presenting different methodological challenges. The different types of databases (GP, claims, and registries) owned by PROTECT partners, also make extrapolation of our findings to wider ranges of data sources possible. Furthermore, our findings will be validated by testing different drug-AE pairs in the same databases and confirmation of drug-AE association in specific registries that include more detailed information on outcomes and potential confounding factors. 

In conclusion, WP2 of PROTECT will assess the influence of methodological parameters on the association between selected AEs and drug class of interest. The selected AEs include resulted in (major) regulatory decisions such as drug withdrawal or SmPC changes or allow the investigation of a broad range of relevant methodological issues. The anticipated results of this project include the creation of a European database network and further development of methodological standards for the conduct of (multi-) national PE studies. Methodological standards will be included when appropriate in the EMA-based ENCePP guidance on methodological standards. Increasing methodological standards and registration of study protocols may decrease discrepancies in results from these studies, increase transparancy and thereby increase the usefulness and reliability of these studies for benefit-risk assessment and decision- making of marketed drugs in Europe and beyond. 

## Figures and Tables

**Table 1.** Selected AE and their characteristics.

AE	Non-Fatal /Fatal Incidence		Regulatory Triggers/Action	Seriousness	Ascertainment	Feasibility of Ascertainment in EHR
**Acute myocardial infarction **	Non-fatal Fatal	803/100,000 hospital discharges due to CHD in 2009 [33]**76 {range: 30-313}/ 100,000 in 2010 [34]	Drug withdrawal/ Boxed warning [30]	10% disability-adjusted life years lost by CHD in 2010 [33] 28-day case fatality of IHD: 34%-88% [35]	Clinical, laboratory and ECG criteria	Moderately Easy
**Idiopathic acute liver injury **	Non-fatal Fatal	1-41/100,000 person years [36-38] 10% of all AE [39] 0.8/million person-years [36]	Drug withdrawal/ Boxed warning [30, 40, 41]	6 months case fatality: 12% [36] 29% of patients acute jaundice [42]	Diverse clinical, laboratory and histological data [43]	Moderately Difficult
**Hip fracture**	Non-fatal Fatal	80-200 /100,000/yr [44] 20-24% fatality rate within 1 yr [45,46]	Warning in product information of antiretrovirals [47] & thiazolidinediones [48, 49]	3.3 years: mean interval between fractures [50]	Hospital admission	Easy
**Suicide/suicide attempt**	Non-fatal Fatal	50-100/100,000/yr attempts [51] 10 /100,000/yr [52]	Drug withdrawal/Boxed warning [30]	-	Cause of death Hospital admission due to self-harm	Difficult
**Cancer**	Non-fatal Fatal	414-600/100,000 new cases/yr [53] 170/100,000/yr [34]	For biologicals [41]	5-year fatality rate: 43%-71% [53]	Tumour diagnosis cancer registry	Moderately Easy

AE = adverse event

IHD = ischemic heart diseases or CHD= coronary heart diseases both terms include acute myocardial infarction

EHR = electronic healthcare records

[ ] = number indicating the reference including these data

**Table 2.** Selected medications and their characteristics.

Drug	Range Prevalence of Drug Exposure per Thousand Inhabitants	Most Frequent Type of Use
**Short / long acting beta-agonists **	66 [54] to 84 [55] /1000	As needed/chronic
**Antimicrobials **	236 [56] to 344 [54] /1000	Short term/long term use
**Antidepressants/benzodiazepines SSRI TCA Benzodiazepines**	30 [56] to 55 [54] /1000 15 [56] to 11 [54] /1000 30 [56] to 81 [54] /1000	As needed/long term use
**Anticonvulsants **	17 [56] to 22 [55] /1000	Chronic
**Calcium channel blockers**	45 [55] to 70 [54] /1000	Chronic

SSRI = selective serotonin reuptake inhibitor

TCA = tricyclic antidepressants

[ ] = number indicating the reference including these data

**Table 3.** Drug–AE associations and characteristics.

	Relative Risk [(RR)		Source (Type of Study)		Main Methodological Issues	Hazard Function
**SABA / LABA and AMI**	RR > 2 for cardiovascular events vs. placebo [57] ORs 1.7 - 7.3 (new users) for MI vs. non-users [57]		Systematic review (RCT) (Case-control)		Protopathic bias Confounding by indication /severity	Acute onset, transient
	RR = 2.5 for respiratory deaths vs. placebo [58]		Meta-analysis (RCT)			
	*salmeterol: *all cause mortality Peto OR =1.3 vs. placebo[59]** non-fatal AE OR = 1.2 vs. placebo [59]* formoterol* : non-fatal serious AE OR = 1.6 vs. placebo[60]		Cochrane database systematic review (RCT)			
	OR 1.2 for beta-2 agonists (current users) – 2.5 (IHD patients) [61]**RR = 1.6 for SABA (heavy users vs. users of <3 months) [62] RR = 1.1 for LABA (heavy users vs. users of <3 months) [62]		Nested case–control cohort			
**Antimicrobials and ALI**	Elevated liver enzymes, cholestasis, and acute liver failure (for betalactam antimicrobials, macrolides, sufonamides, tetracyclines [63]		Case reports/ retrospective cohort		Definition/measurement of the outcome Ascertaining/tracing of exposure (short time window)	Acute/intermediate onset (3-4 weeks) after drug stop
	RRs 2.3 (Amoxicillin without clavulanic acid) – 1299.9 (Isoniazid + rifampicin + pyrazinamide) [64]		Case-population			
	ORs 5.3 (erythromycin) – 94.8 (amoxicillin/clavulanic acid) [37]		Case-control (pop-based)			
**Antidepressants/ BZD and hip fracture**	RRs 1.2 - 3.7 for TCA users [65] RRs 1.5 - 8.6 for SSRI users [65] RRs 1.5 - 2.0 for hypnotics including BZD [66]		Case-control/ cohort		Exposure classification (for antidepressants) Selection bias Unmeasured confounding	SSRIs: peak at 6–12 months [67] TCA’s: peak at 1-2 months [67] BZD: acute
**Anticonvulsants and suicide/-attempts**	RR = 2 for 11 different groups of the drug (1.5 (psychiatric) 3.5 (epilepsy) risk by indication) [68]		Meta-analysis of RCT		Definition and measurement of outcome	Acute
	RR = 3.1 for current users (lamotrigine, gabapentin, ethosuximide, vigabatrin) [69] OR 2.57 vs. non-users [70]		Nested case-control			
	HRs 1.4 – 2.4 vs. topiramate users [71]		Cohort			
**CCB and cancer**	RRs 1.7 (vs. non-users) - 2.6 (breast cancer) [72, 73] RR = 2.1 for verapamil [74]		Cohort		Long latent period Selection bias Unmeasured confounding	Long-term, delayed

SABA = short acting beta-2 agonists

LABA = long acting beta-2 agonists

(A)MI = (acute) myocardial infarction

ALI = acute liver injury

BZD = benzodiazepines

SSRI = selective serotonin reuptake inhibitor

RR = relative risk; OR =odds ratio, HR= hazard ratio

IHD = Ischemic Heart Diseases

AE = adverse event

TCA = tricyclic antidepressants

[Number] = number of reference including these data

CCB = calcium channel blockers

**Table 4.** Characteristics of participating databases.

Database / Country	Danish Registries (DK)	Mondriaan (NL)	GPRD (UK)	THIN (UK)	BIFAP (ES)	Bavarian Claims (DE)
**Nr. of persons with historical data (in Millions)**	approx. 6	1.4 (GP) 13.5 (pharmacy) 1.2 (claims)	11.2	11	3.2	10.5
**Nr. of active persons in 2008 (in millions)**	5.2	0.6	4.6	3.8	1.6	9.5
**Starting year of data collection**	1994 a 1977 b	1991	1987	2003	2001	2001
**Nationwide**	+	90% of NL (pharmacy)	7% of the UK	6.2% of the UK	7% of Spain	
**Representative of nation**	+	+	+	+	+e	+c
**Type of database**						
General practitioner	+	+	+	+	+	+h
Pharmacy	+	+	/ f		/ f	+h
Mortality registry	+	/ linkage	+ g		/	
Cancer registry	+		+ linkage			
Hospitalisation registry	+	/linkage	+ linkage		/	
Specialist/secondary care	+	/	+ linkage			+
Claims	+	+				+
National statistics	+		/			
Surveys		+				
Routine data quality checks	+	+	+	+	+	+
Possibility of prospective data collection among patients in the database d		/	+	+	+	

DK = Denmark, NL = The Netherlands, UK = United Kingdom, ES = Spain, DE = Germany

+ = data is available

/ = data is partly available

a = Medicinal products

b = Patient registration

c = representative of the region

d = For Interviews, trials, surveys

e = GPs from 9 out of 17 regions in Spain. 15% of the collaborating regions and 7% of the total population. Representative of population attending primary care in Spain (similar age and sex distribution)

f = prescribed not dispensed

g = contains records of death but is not the official registry

h = prescriptions and diagnoses are only available per quarter (no exact dates)

## References

[1] Strom BL, Kimmel SE, Hennessy S (2012). Pharmacoepidemiology.. 5th ed. Wiley-Blackwell: Oxford.

[2] Strom BL (1995). Pharmacoepidemiology: Response to a challenge.. Pharmacoepidemiol Drug Saf.

[3] Horwitz RI, Feinstein AR (1980). The problem of "protopathic bias" in case-control studies.. Am J Med.

[4] Lewis MA (1998). The epidemiology of oral contraceptive use: a critical review of the studies on oral contraceptives and the health of young women.. Am J Obstet Gynecol.

[5] Taubes G (1995). Epidemiology faces its limits.. Science.

[6] Klungel OH, Martens EP, Psaty BM  (2004). Methods to assess intended effects of drug treatment in observational studies are reviewed.. J Clin Epidemiol.

[7] (2013). CPRD The UK Clinical Practice Research Datalink.. http: //www.cprd.com/intro.asp. Accessed December 11.

[8] Meier CR, Schlienger RG, Kraenzlin ME, Schlegel B, Jick H (2000). HMG-CoA reductase inhibitors and the risk of fractures.. JAMA.

[9] van Staa TP, Wegman S, de Vries F, Leufkens B, Cooper C (2001). Use of statins and risk of fractures.. JAMA.

[10] de Vries F, de Vries C, Cooper C, Leufkens B, van Staa TP (2006). Reanalysis of two studies with contrasting results on the association between statin use and fracture risk: the General Practice Research Database.. Int J Epidemiol.

[11] Guess HA (2006). Exposure-time-varying hazard function ratios in case-control studies of drug effects.. Pharmacoepidemiol Drug Saf.

[12] Klungel OH, Stricker BH, Breteler MM, Seidell JC, Psaty BM, de Boer A (2001). Is drug treatment of hypertension in clinical practice as effective as in randomized controlled trials with regard to the reduction of the incidence of stroke?.. Epidemiology.

[13] Martens EP, de Boer A, Pestman WR, Belitser SV, Stricker BH, Klungel OH (2008). Comparing treatment effects after adjustment with multivariable Cox proportional hazards regression and propensity score methods.. Pharmacoepidemiol Drug Saf.

[14] Martens EP, Pestman WR, de Boer A, Belitser SV, Klungel OH (2008). Systematic differences in treatment effect estimates between propensity score methods and logistic regression.. Int J Epidemiol.

[15] Suissa S (2007). Immortal time bias in observational studies of drug effects.. Pharmacoepidemiol Drug Saf.

[16] Hubbard RB, Smith CJ, Smeeth L, Harrison TW, Tattersfield AE (2002). Inhaled corticosteroids and hip fracture: a population-based case-control study.. Am J Respir Crit Care Med.

[17] van Staa TP, Leufkens B, Cooper C (2002). Bone loss and inhaled glucocorticoids.. N Engl J Med.

[18] Schlienger RG, Kraenzlin ME, Jick SS, Meier CR (2004). Use of beta-blockers and risk of fractures.. JAMA.

[19] de Vries F, Souverein PC, Cooper C, Leufkens HG, van Staa TP (2007). Use of beta-blockers and the risk of hip/femur fracture in the United Kingdom and The Netherlands.. Calcif Tissue Int.

[20] Cardwell CR, Abnet CC, Cantwell MM, Murray LJ (2010). Exposure to oral bisphosphonates and risk of esophageal cancer.. JAMA.

[21] Green J, Czanner G, Reeves G, Watson J, Wise L, Beral V Oral bisphosphonates and risk of cancer of oesophagus.. stoach.and colorectum case control analysis within a UK primary care cohort.

[22] Yang YX, Lewis JD, Epstein S, Metz DC (2006). Long-term proton pump inhibitor therapy and risk of hip fracture.. JAMA.

[23] Kaye JA, Jick H (2008). Proton pump inhibitor use and risk of hip fractures in patients without major risk factors.. Pharmacotherapy.

[24] de Vries F, van Staa TP, Leufkens HG ( 2010). Proton pump inhibitors. fracture risk and selection bias: three stuies.same database., two answers.. Osteoporos Int.

[25] de Vries F, Cooper AL, Cockle SM, van Staa TP, Cooper C (2009). Fracture risk in patients receiving acid-suppressant medication alone and in combination with bisphosphonates.. Osteoporos Int.

[26] (2013). Pharmacoepidemiological Research on Outcomes of Therapeutics by a European Consortium (PROTECT).. http: //www.imi-protect.eu. Accessed December 11.

[27] (2013). Foundation for the National Institutes of Health.. Observational Medical Outcomes Partnership (OMOP). http: //omop.fnih.org. Accessed December 11.

[28] (2013). European Network of Centres for Pharmacoepidemiology and Pharmacovigilance.. http//: www.encepp.eu. Accessed December 11.

[29] (2013). The US Food and Drug Administration.. FDA's Sentinel Initiative. http: //www.fda.gov/Safety/FDAsSentinelInitiative. Accessed December 11.

[30] Trifiro G, Pariente A, Coloma PM  (2009). Data mining on electronic health record databases for signal detection in pharmacovigilance: which events to monitor?.. Pharmacoepidemiol Drug Saf.

[31] Stang PE, Ryan PB, Racoosin JA  (2010). Advancing the science for active surveillance: rationale and design for the Observational Medical Outcomes Partnership.. Ann Intern Med.

[32] Grimaldi-Bensouda L, Rossignol M, Aubrun E, Benichou J, Abenhaim L (2012). Agreement between patients' self-report and physicians' prescriptions on nonsteroidal anti-inflammatory drugs and other drugs used in musculoskeletal disorders: the international Pharmacoepidemiologic General Research eXtension database.. Pharmacoepidemiol Drug Saf.

[33] (2013). European Heart Network.. New European Cardiovascular Disease Statistics 2012. http: //www.ehnheart.org/cvd-statistics. Accessed December 11.

[34] (2013). European Commission Eurostat.. Statistics Public Health: main tables. http: //epp.eurostat.ec.europa/tgm/table.do?..tab=table&init=1&
language=en&pcode=tps00119&plugin=1. Accessed December 11.

[35] (2013). Health in Europe: Information and Data Interface: cardiovascular diseases https: //webgate.. c.europa.eu/sanco/heidi/index.php/Heidi/
Major_and_chronic_diseases/Cardiovascular_disease/Ischaemic_heart_disease. Accessed December 11.

[36] Ibanez L, Perez E, Vidal X, Laporte JR (2002). Prospective surveillance of acute serious liver disease unrelated to infectious. obstrucive.or metabolic diseases epidemiological and clinical features., and exposure to drugs.. J Hepatol.

[37] de Abajo FJ, Montero D, Madurga M, Garcia Rodriguez LA (2004). Acute and clinically relevant drug-induced liver injury: a population based case-control study.. Br J Clin Pharmacol.

[38] Sgro C, Clinard F, Ouazir K  (2002). Incidence of drug-induced hepatic injuries: a French population-based study.. Hepatology.

[39] Shapiro MA, Lewis JH (2007). Causality assessment of drug-induced hepatotoxicity: promises and pitfalls.. Clin Liver Dis.

[40] Lexchin J (2005). Drug withdrawals from the Canadian market for safety reasons.. 1963-2004. CMAJ.

[41] Giezen TJ, Mantel-Teeuwisse AK, Straus SM, Schellekens H, Leufkens HG, Egberts AC (2008). Safety-related regulatory actions for biologicals approved in the United States and the European Union.. JAMA.

[42] Vuppalanchi R, Liangpunsakul S, Chalasani N (2007). Etiology of new-onset jaundice: how often is it caused by idiosyncratic drug-induced liver injury in the United States?.. Am J Gastroenterol.

[43] Slavin DE, Schlichting CL, Freston JW (2005). Rating the severity of the medical consequences of drug-induced liver injury.. Regul Toxicol Pharmacol.

[44] (2004). International Osteoporosis Foundation.. Osteoporosis in Europe: Indicators of Progress Outcomes of panel meeting 2005 No 10.http: //www.iofbonehealth.org/osteoporosis-europe-inbdicators-progress-2005.

[45] Cooper C, Atkinson EJ, Jacobsen SJ, O'Fallon WM, Melton LJ3rd (1993). Population-based study of survival after osteoporotic fractures.. Am J Epidemiol..

[46] Leibson CL, Tosteson AN, Gabriel SE, Ransom JE, Melton LJ (2002). Mortality disability and nursing home use for persons with and without hip fracture: a population-based study.. J Am Geriatr Soc..

[47] (2013). European Medicines Agency.. European Public Assessment Reports (EPAR). antiretroviral: http: //www.ema.europa.eu/docs/en_GB/
document_library/EPAR_-_Product_Information/human/000797/
WC500028102.pdf. Accessed December 11..

[48] (2013). European Medicines Agency.. European Public Assessment Reports (EPAR). pioglitazone: http: //www.ema.europa.eu/docs/en_GB/
document_library/Other/2011/07/WC500109185.pdf. Accessed December 11..

[49] (2013). European Medicines Agency.. Summary of product charactersitics rosiglitazone: http: //www.ema.europa.eu/docs/en_GB/document_
library/EPAR_-_Product_Information/human/000268/WC00029108.
pdf. Accessed December 11..

[50] Schroder HM, Petersen KK, Erlandsen M (1993). Occurrence and incidence of the second hip fracture.. Clin Orthop Relat Res..

[51] ( 2013). Statistics Netherlands: http: //statline.bs.nl/StatWeb/publication/?.DM=SLNL&PA =71859NED&1=1..,5.,7-8&D2=1-2&D3=1&
D4=189&D5=l&VW=T. Accessed December 11..

[52] (2013). European Commission EUROSTAT.. Death due to suicide by gender 2010 November 2012: http: //epp.eurostat.ec.europa/tgm/
table.do?..tab=table&init=1language=en&pcode= tps00122&plugin=1. Accessed December 11..

[53] (2013). Health in Europe Information and Data Interface Cancer.. http: //webgate.ec.europa.eu/sanco/heidi/index.php/Heidi/Major_and_chronic_diseases/ Cancer#Adult_cancer_survival. Accessed December 11..

[54] (2013). Statens Serum Institut.. Register of Medicinal Product Statistics. http: //www.medstat.dk/en. Accessed December 11..

[55] (2013). Norwegen Prescription Database.. http: //www.norpd.no/prevalens.aspx. Accessed December 11..

[56] (2013). GIPdatabank The Drug Information System of the Health Care Insurance Board The Netherland: http: //www.ipdatabank.nl. Accessed December 11..

[57] Salpeter SR (2004). Cardiovascular safety of beta(2)-adrenoceptor agonist use in patients with obstructive airway disease a systematic review.. Drugs Aging..

[58] Salpeter SR, Buckley NS, Salpeter EE (2006). Meta-analysis anticholinergics but not beta-agonists reduce severe exacerbations and respiratory mortality in COPD.. J Gen Intern Med..

[59] Cates CJ, Lasserson TJ, Jaeschke R (2009). Regular treatment with salmeterol and inhaled steroids for chronic asthma serious adverse events.. Cochrane Database Syst Rev..

[60] Cates CJ, Lasserson TJ, Jaeschke R (2009). Regular treatment with formoterol and inhaled steroids for chronic asthma serious adverse events.. Cochrane Database Syst Rev..

[61] de Vries F, Pouwels S, Bracke Metal (2008). Use of beta2 agonists and risk of acute myocardial infarction in patients with hypertension.. Br J Clin Pharmacol..

[62] Zhang B, de Vries F, Setakis E, van Staa TP (2009). The pattern of risk of myocardial infarction in patients taking asthma medication a study with the General Practice Research Database.. J Hypertens..

[63] Neil Kaplowitze LDD (2003). Drug-induced Liver Disease 1 ed.. Informa Healthcare..

[64] Sabate M, Ibanez L, Perez Eetal (2007). Risk of acute liver injury associated with the use of drugs a multicentre population survey.. Aliment Pharmacol Ther..

[65] Ginzburg R, Rosero E (2009). Risk of fractures with selective serotonin-reuptake inhibitors or tricyclic antidepressants.. Ann Pharmacother..

[66] Allain H, Bentue-Ferrer D, Polard E, Akwa Y, Patat A (2005). Postural instability and consequent falls and hip fractures associated with use of hypnotics in the elderly a comparative review.. Drugs Aging..

[67] (2013). MHRA Medicines and Healthcare products Regulatory Agency.. Drug Safety Update Antidepressants risk of fractures 2010. http: //www.mhra.gov.uk/Safetyinformation/DrugSafetyUpdate/CON085136. Accessed December 11..

[68] (2013). US Food and Drug Agency.. Statistical Review and Evaluation Antiepleptic Drugs and Suicidality. http: //www.fda.gov/downloads/
Drugs/DrugSafety/PostmarketDrugSafetyInformationd for Patients and
Providers/UCM192556.pdf. Accessed December 11..

[69] Andersohn F, Schade R, Willich SN, Garbe E (2010). Use of antiepileptic drugs in epilepsy and the risk of self-harm or suicidal behavior.. Neurology..

[70] Arana A, Wentworth CE, Ayuso-Mateos JL, Arellano FM (2010). Suicide-related events in patients treated with antiepileptic drugs.. N Engl J Med..

[71] Patorno E, Bohn RL, Wahl PMetal (2010). Anticonvulsant medications and the risk of suicide attempted suicide or violent death.. JAMA..

[72] Pahor M, Guralnik JM, Ferrucci Letal (1996). Calcium-channel blockade and incidence of cancer in aged populations.. Lancet..

[73] Fitzpatrick AL, Daling JR, Furberg CD, Kronmal RA, Weissfeld JL (1997). Use of calcium channel blockers and breast carcinoma risk in postmenopausal women.. Cancer..

[74] Beiderbeck-Noll AB, Sturkenboom MC, van der Linden PDetal (2003). Verapamil is associated with an increased risk of cancer in the elderly the Rotterdam study.. Eur J Cancer..

